# Kinetic and X‐ray Structural Characterization of Phospholipase C Gamma 2 PLCG2–an Emerging Target for Alzheimer’s Disease

**DOI:** 10.1002/alz.095639

**Published:** 2025-01-09

**Authors:** Bridget L Kaiser, Emma K Lendy, Nicolas Morato, Yunfei Feng, Daniel E Beck, Karson S Putt, Graham Cooks, Andrew D. Mesecar

**Affiliations:** ^1^ Purdue, West Lafayette, IN USA; ^2^ Indiana Biosciences Research Institute, Indianapolis, IN USA; ^3^ Purdue University, West Lafayette, IN USA

## Abstract

**Background:**

PLCG2 is signal‐transduction protein identified as a potential drug target for the treatment of Alzheimer’s disease (AD). PLCG2 is regulated by stimulation of the TREM2 pathway in microglia, which results in phagocytosis of beta‐amyloid. PLCG2 catalyzes the cleavage of PI(4,5)P2 into IP3 and diacylglycerol, resulting in increased cell motility, phagocytosis, and proliferation in microglia. Studies found that a naturally occurring PLCG2 variant, P522R, stimulates PLCG2 activity and is associated with decreased AD risk, making PLCG2 an appealing target as an AD therapeutic. Current drug discovery efforts for this enzyme are limited by no publicly available structures of full‐length PLCG2 and limited kinetic characterization studies. In this study, kinetic and structural studies of wild‐type and the P522R variant of PLCG2 are explored and presented.

**Method:**

PLCG2 was expressed and purified from baculovirus infected Sf9 cells for in‐vitro studies. Kinetic characterization of PLCG2 was accomplished using a continuous fluorescence‐based assay and a mass spectrometry‐based end‐point assay called Desorption Ionization Mass Spectrometry (DESI‐MS). Structural studies of PLCG2 wild‐type and P522R variant were pursued using single‐particle cryo‐EM and X‐ray crystallography.

**Result:**

Kinetic parameters were quantified with both the fluorescence and label‐free DESI assays and found to be similar between assays. X‐ray data on wild‐type PLCG2 crystals were collected to 2.8Å. The current X‐ray structural model reveals an intact active site with bound calcium atom and first‐sphere waters, but a missing nSH2 domain (no observable electron density). Cryo‐EM maps of wild‐type and P522R are consistent with the X‐ray structural model including poor density for the nSH2 domain. Comparison of human PLCG2 structural models to AlphaFold and rat PLCG1 models reveals differences in domain xSH2 domain positions.

**Conclusion:**

Human PLCG2 is highly active in solution as determined by kinetic studies using two different biochemical assays. The X‐ray and cryo‐EM structures of PLCG2 reveal significant conformational flexibility of the nSH2 domain which may be an important function in it binding to other proteins in the plasma membrane.

This research was supported by grant 1U54 AG065181 (IUSM‐Purdue TREAT‐AD Center) from the National Institute of Aging, National Institutes of Health.

